# Supplementary material for: Mediating Effects of Diagnostic Route on the Comorbidity Gap in Survival of Patients with Diffuse Large B-Cell or Follicular Lymphoma in England
Source: Cancers (Basel). 2022 Oct 17;14(20):5082. doi: 10.3390/cancers14205082 (PMC9599821; doi:10.3390/cancers14205082)
Supplement: Supplementary file 1 [file cancers-14-05082-s001.zip › cancers-1906549-supplementary.pdf]

Supplementary Table S1: Comorbidities and their diagnostic ICD-10 codes

Comorbidity	ICD-10
Myocardial infarction	I21.x, I22.x, I25.2
Congestive cardiac failure	I11.0, I13.0, I13.2, I25.5, I42.0, I42.5–I42.9, I43.x, I50.x, P29.0
Peripheral vascular disease	I70.x, I71.x, I73.1, I73.8, I73.9, I77.1, I79.0, I79.2, K55.1, K55.8, K55.9, Z95.8, Z95.9
Cerebrovascular disease	G45.x, G46.x, H34.0, I60.x–I69.x
Dementia	F00.x–F03.x, F05.1, G30.x, G31.1
Chronic obstructive pulmonary disease	I27.9, J40.x–J47.x, J60.x–J67.x, J68.4, J70.1, J70.3
Rheumatological disease	M05.x, M06.x, M31.5, M32.x–M34.x, M35.1, M35.3, M36.0
Liver disease	B18.x, K70.0–K70.3, K70.9, K71.3–K71.5, K71.7, K73.x, K74.x, K76.0, K76.2–K76.4, K76.8, K76.9, Z94.4, K71.1, K72.1, K72.9, K76.5, K76.6, K76.7, I85.0, I85.9, I86.4, I98.2, K70.4,
Diabetes mellitus	E10.0 – E14

Hemiplegia or paraplegia	G04.1, G11.4, G80.1, G80.2, G81.x, G82.x, G83.0–G83.4, G83.9
Renal disease	I12.0, I13.1, N03.2–N03.7, N05.2–N05.7, N18.x, N19.x, N25.0, Z49.0–Z49.2, Z94.0, Z99.2
AIDS/HIV	B20.x–B22.x, B24.x

ICD-10: International Classification of Diseases, 10th Revision

Diabetes with/without chronic complication is combined within the RCS Charlson Comorbidity Score

Supplementary Table S2. Distribution of non-Hodgkin lymphoma subtypes for patients diagnosed from 2005-2013, with respective morphology and topography ICD-O-3 codes.

Index	Site group (subtype)	Progression	Topography	Morphology	n	%
1	CLL/SLL*	Indolent	C80.0-C85.9	9670, 9823	4,043	4.78
2	Waldenstrom macroglobulinemia	Indolent	C80.0-C85.9	9761	2,453	2.90
3	Mantle cell	Indolent	C80.0-C85.9	9673	3,549	4.20
4	Diffuse large B-cell	Aggressive	C80.0-C85.9	9680, 9688, 9737-9738	30,750	36.39
5	Burkitt	Aggressive	C80.0-C85.9	9687, 9826	1,077	1.27
6	Follicular	Indolent	C80.0-C85.9	9690-9691, 9695, 9698	15,624	18.49
7	Mature T-cell	Aggressive	C80.0-C85.9	9702	6,066	7.18
8	Marginal zone B-cell	Indolent	C80.0-C85.9	9689, 9699, 9760, 9764, 9699	4,615	5.46
9	Not Otherwise Specified	n/a	C80.0-C85.9	9591, 9675, 9735	10,308	12.20
10	Other***	n/a	C80.0-C85.9	9591, 9675, 9735	6,019	7.12
Total					84,504	100.00**

n/a – not applicable; there was no subtype information

* Chronic lymphocytic leukaemia/Small-cell lymphocytic lymphoma

** Percentages may not equate to 100.0% due to rounding

*** The morphology code specifies these patients are diagnosed with NHL. However, the description states ‘other’; these patients are classified similarly to ‘Not Otherwise Specified’.
